# Identification of Biomarkers of Impaired Sensory Profiles among Autistic Patients

**DOI:** 10.1371/journal.pone.0164153

**Published:** 2016-11-08

**Authors:** Afaf El-Ansary, Wail M. Hassan, Hanan Qasem, Undurti N. Das

**Affiliations:** 1 Central Laboratory, Center for Female Scientific and Medical Colleges, King Saud University, Riyadh, Saudi Arabia; 2 Biochemistry Department, Science College, King Saud University, Riyadh, Saudi Arabia; 3 Therapuetic Chemistry Department, National Research Centre, Dokki, Cairo, Egypt; 4 Department of Biomedical Sciences, College of Health Sciences, University of Wisconsin – Milwaukee, Milwaukee, Wisconsin, United States of America; 5 UND Life Sciences, 2020 S 360^th^ St, # K-202, Federal Way, Washington, 98003, United States of America; 6 BioScience Research Centre, GVP College of Engineering Campus, Visakhapatnam-530048, India; Texas Technical University Health Sciences Center, UNITED STATES

## Abstract

**Background:**

Autism is a neurodevelopmental disorder that displays significant heterogeneity. Comparison of subgroups within autism, and analyses of selected biomarkers as measure of the variation of the severity of autistic features such as cognitive dysfunction, social interaction impairment, and sensory abnormalities might help in understanding the pathophysiology of autism.

**Methods and Participants:**

In this study, two sets of biomarkers were selected. The first included 7, while the second included 6 biomarkers. For set 1, data were collected from 35 autistic and 38 healthy control participants, while for set 2, data were collected from 29 out of the same 35 autistic and 16 additional healthy subjects. These markers were subjected to a principal components analysis using either covariance or correlation matrices. Moreover, libraries composed of participants categorized into units were constructed. The biomarkers used include, PE (phosphatidyl ethanolamine), PS (phosphatidyl serine), PC (phosphatidyl choline), MAP2K1 (Dual specificity mitogen-activated protein kinase kinase 1), IL-10 (interleukin-10), IL-12, NFκB (nuclear factor-κappa B); PGE2 (prostaglandin E2), PGE2-EP2, mPGES-1 (microsomal prostaglandin synthase E-1), cPLA2 (cytosolic phospholipase A2), 8-isoprostane, and COX-2 (cyclo-oxygenase-2).

**Results:**

While none of the studied markers correlated with CARS and SRS as measure of cognitive and social impairments, six markers significantly correlated with sensory profiles of autistic patients. Multiple regression analysis identifies a combination of PGES, mPGES-1, and PE as best predictors of the degree of sensory profile impairment. Library identification resulted in 100% correct assignments of both autistic and control participants based on either set 1 or 2 biomarkers together with a satisfactory rate of assignments in case of sensory profile impairment using different sets of biomarkers.

**Conclusion:**

The two selected sets of biomarkers were effective to separate autistic from healthy control subjects, demonstarting the possibility to accurately predict the severity of autism using the selected biomarkers. The effectiveness of the identified libraries lied in the fact that they were helpful in correctly assigning the study population as control or autistic patients and in classifying autistic patients with different degree of sensory profile impairment.

## Introduction

Autism is a heterogeneous neurodevelopmental disorder in which there exists a complex interaction (s) between genetic and environmental risk factors. Autism is characterized by social interaction impairment, repetitive behavior and sensory abnormalities [1,2] and is an important cause of childhood disability that imposes significant burden on the parents and society [3,4]. Autism is a spectrum of disorders, implying that the clinical phenotype can present in a variety of combinations with a high degree of variability especially in the severity of cognitive manifestations, that can range from a non-verbal child with mental retardation to a those who possess above average IQ with inadequate social skills [5,6]. This heterogeneity of autism presents significant challenges to identify biochemical correlates that might help in the early diagnosis [7]. Hence, identifying valid and reliable biomarkers that are less prone to error to diagnose autism is urgently needed. Current practice to arrive at a diagnosis of Autism Spectrum Disorder (ASD) requires a multi-method approach that includes: observation of the child, caregiver interview, assessment of developmental levels, detailed developmental history, and screening for associated disorders such as Fragile X [8]. Several ASD screening and diagnostic procedures that were developed lately include: childhood autism rating scale (CARS) [9], social responsiveness scale (SRS) [10] and short sensory profile (SSP) [11]. It is likely that a better understanding of the heterogeneity of autism could generate useful information that may aid in understanding and study of its etiology, diagnosis, treatment and prognosis [12]. Impairment in sensory processing (SP) has been reported in 42% to 88% of children with autism; however, observational research examining the existence sensory processing dysfunction within autistic children is rare. Furthermore, little attention has been given to examining the relationship between sensory processing dysfunction and the biomarkers that are measured in autistic patients [13,14,15,16,17].

Several candidate biomarkers are emerging as promising biomarkers of autism and may pave way in elucidating its biological understanding. It is also possible that development of reliable and robust biomarkers may facilitate early identification, personalized treatment, and improved outcomes of autism. In the present study, we have tested two sets of markers by PCA (principle component analysis): the first batch consisted of phosphatidyl ethanolamine (PE), phosphatidylserine (PS), phosphatidylcholine (PC), mitogen-activated protein kinase kinase 1 (MAP2K1), interleukin 10 (IL-10), interleukin 12 (IL-12) and nuclear factor kappa B (NF-κB) and the second batch included markers related to lipid signaling: (COX-2/PGE2) pathway represented by prostaglandin E2 (PGE2), prostaglandin E2 receptor 2 (PGE2-EP2), microsomal prostaglandin E synthase 1 (mPGES-1), cyclooxygenase-2 (COX-2), cytosolic phospholipase A2 (cPLA2) and 8-Isoprostane. We studied SP patterns and SSP in children with autistic disorder and correlated the same with the aforementioned biomarkers.

## Methods

### 2.1 Participants

The study protocol was approved by the ethics committee of medical Collage, King Saud University according to the most recent Declaration of Helsinki (Edinburgh, 2000). Two batches of study populations were recruited for the study consisting of 35 autistic patients and 38 age and gender matched healthy control in the first group and the second group included 29 autistic patients and 16 control participants (Disparity in the number of participants is due to the insufficiency of the collected plasma samples of the second batch). All participants gave written informed consent provided by their parents and agreed to participate in the study. The study participants were enrolled in the study through the ART Center (Autism Research & Treatment Center) clinic. The ART Center clinic sample population consisted of children diagnosed with ASD. The diagnosis of ASD was confirmed in all study subjects using the Autism Diagnostic Interview-Revised (ADI-R) and the Autism Diagnostic Observation Schedule (ADOS) and 3DI (Developmental, dimensional diagnostic interview) protocols. The ages of autistic children included in the study were between 2–12 years old. All were simplex cases. All are negative for fragile x gene study. The control group was recruited from pediatric clinic at King Saud medical city whose mean age ranged from 2–14 years. Subjects were excluded from the investigation if they had dysmorphic features, or diagnosis of fragile X or other serious neurological (e.g., seizures), psychiatric (e.g., bipolar disorder) or known medical conditions. All participants were screened via parental interview for current and past physical illness. Children with known endocrine, cardiovascular, pulmonary, liver, kidney or other medical disease were excluded from the study. All patients and controls included in the study were on similar but not identical diet and none of them were on any special high fat or fat restricted diet.

#### 2.1.1Behavioral assessment

The Short Sensory Profile is a 38-item questionnaire intended to rate a variety of sensory impairments. Each item on the SSP is measured on a 5-point Likert scale. Domain scores were measured in the areas of tactile sensitivity, taste/smell sensitivity, movement sensitivity, seeking sensation, auditory filtering, low energy levels, and visual/auditory sensitivity. Domain scores and overall sensory response were categorized as typical performance, probable difference from typical performance, or definite difference from typical performance. Scores less than 142 indicate severe performance (definite difference from typical performance), scores between 142 and 152 indicate mild to moderate performance (probable difference from typical performance) and scores between 153 and 190 indicate typical performance. The SSP provides information about sensory processing skills of children with autism and assists occupational therapists in assessing and planning much needed interventions for these children [11,18]

### 2.2 Sample collection

After overnight fasting, blood samples were collected from autistic children and healthy controls from the antecubital vein of the arm by a qualified lab technician into 3-ml blood collection tubes containing EDTA. Immediately after collection, blood was centrifuged at 4°C at 3000 g for 20 minutes. The plasma was decanted, dispensed into four 0.75 ml aliquots (to avoid multiple freeze-thaws cycles) and stored at −80°C until analysis.

## Ethics approval and consent

This work was approved by the ethics committee of King Khalid Hospital, King Saud University (Approval number: 11/2890/IRB). A written consent was obtained from the parents of all participants recruited in the study as per the guidelines of the ethics committee.

## Biomarkers selection and measurements

The selected biomarkers were measured in the plasma samples of both autistic patients and control. After initial assessment of the overall discriminatory power of 19 biomarkers through its maximal area under the curve (AUC), as the best discriminatory power that the biomarker can achieve, the presented 13 biomarkers were selected based on their recorded satisfactory (AUC), specificity and sensitivity when analyzed using receiver operating characteristics. A brief description of the biochemical indices measured is summarized below.

Phospholipid extraction was done performed using chloroform and methanol (1:3 v/v) solvents system. PC, PS, PE were measured in the extract by HPLC Bligh and Dyer procedure (1959)[19]. Standard PC, PS and PE were obtained from Fluka, Sigma-Aldrich (Taufkirchen, Germany). IL-10 and IL-12 was measured using Invitrogen human product, a solid phase sandwich ELISA tests according to the manufacturer’s protocol. NF-kB was measured using ELISA kit obtained from Oxford Biomedical Research, USA. This method is based on the use of oligonucleotide, containing the DNA binding NF-kB consensus sequence, bound to a 96 –well plate. MAP2K1 was measured using a Sandwich enzyme immunoassay ELISA kit, product of USCN (Uscn Life Science Inc. Wuhan). 8-isoprostane was measured using a non-radioactive, ELISA kit obtained from My BioSource (My BioSource, Inc San Diego, CA, 92195–3308, USA) COX-2 was measured by ELISA kit obtained from CUSABIO (8400 Baltimore Avenue, Room 332 College Park, MD 20740). cPLA2 was measured using a competitive enzyme immunoassay kit that utilizes a monoclonal anti-cPLA2 antibody and a cPLA2-HRP conjugate obtained from Amsbio (184 Park Drive, Milton Park, Abingdon OX14 4SE, UK). MPGES-1 was measured Elisa kit obtained from Wuxi Donglin Sci & Tech Development (A3-South,15# Shuigoutou,Wuxi/Jiangsu Province, PRC). PGE2 and PGE2-EP2 were measured by Elisa kits obtained from Uscn, (Life Science Inc, USA).

## Statistical analysis

Principal component analysis (PCA) was done using either BioNumerics version 6.6 (Applied Maths, Austin, Texas) or IBM SPSS version 22 (IBM Corporation, Armonk, NY). PCA was performed using either covariance or correlation matrices. Whenever, a covariance matrix was used, values were divided by the variances of their respective variables to normalize weighting of variables (i.e. to avoid variables with smaller numbers being obscured by ones with large values). Bartlett’s sphericity test and Kaiser-Meyer-Olkin (KMO) measure of sampling adequacy were done to evaluate the appropriateness of using PCA. Since PCA is a data reduction technique that reduces the number of variables by condensing correlated variables into principal components, it is only appropriate to use PCA in the presence of correlated variables. In the absence of any correlation between variables, those variables are said to be orthogonal and their correlation matrix is an identity matrix (i.e. a matrix composed of “1”s in the diagonal and “0”s everywhere else). Therefore, the null hypothesis—that there is no correlation between any of the variables—is upheld whenever the variables’ correlation matrix is an identity matrix, and is rejected when the probability of the correlation matrix being an identity matrix is shown to be remote. In the present study, Bartlett’s sphericity test was used to reject the null hypothesis that the correlation matrix is identical to the identity matrix and a p value ≤ 0.05 was accepted. Kaiser-Meyer-Olkin (KMO) measure of sampling adequacy compares the correlations to partial correlations between variables. IN this study, KMO values of ≥ 0.7 were accepted. Both Bartlett’s sphericity test and KMO measure of sampling adequacy were done using IBM SPSS. The number of statistically significant components to be extract when performing PCA was determined using Parallel Analysis—also known as Monte Carlo simulation—using Brian O’Connor’s syntax for SPSS [20]. In this study, 1000 permutations (datasets) and eigenvalues were computed for 50^th^ and 95^th^ percentiles. The eigenvalues obtained from PCA were compared to the eigenvalues generated from Monte Carlo simulation in screen plot. Components were considered statistically significant when they had higher eigenvalues compared to the corresponding simulated 95^th^ percentile values. Multidimensional scaling (MDS), cluster analysis, and ANOVA were done using BioNumerics. In ANOVA, the null hypothesis was assessed using Wilks’ lambda likelihood ratio F-test (multivariate) and Fisher’s F-test (univariate). Dendrograms (trees) were constructed from Canberra distances eq (1) using neighbor joining algorithm. Correlations were calculated using Pearson or Spearman correlations using GraphPad Prism version 6 (GraphPad Software, Inc., La Jolla, CA). Multiple regressions using the stepwise method were done using IBM SPSS.

### Library identification

Libraries composed of participants categorized into units were constructed. Individual participants were sequentially removed from the library and submitted for identification. K-nearest neighbor algorithm was used to match participants to library units. In this algorithm, a participant submitted for identification is compared to all library entries (i.e. participants) and the most similar entries are identified. The number of the most similar entries is arbitrarily determined by the user, but has to be equal to or less than the number of entries in the smallest library unit. The unknown is then assigned to the unit that contains the highest number of the most similar entries. In addition, a score is calculated for each identified participant by comparing the number of matched entries to the total number in the library unit. A score of 100 is obtained when all the top matching entries belong to the same unit, while a score of 50 in the case of a two-unit library is obtained if entities were equally divided between the two library units. Higher scores suggest higher level of confidence in the identification process. Similarity values in the current study were calculated from raw biomarker data using Canberra distances eq (1).

$$D=\frac{1}{n}\sum_{i=1}^{n}\frac{\left|Xi-Yi\right|}{\left|Xi+Yi\right|}$$ (1)

Where: “*D*” is the Canberra distance metric, “*n*” is the number of variables, “*i*” is the i^th^ variable, and “*X*” and “*Y*” are two subjects.

## Results

### Autistic and healthy control subjects are effectively separated based on two sets of biomarkers

**Fig 1:**
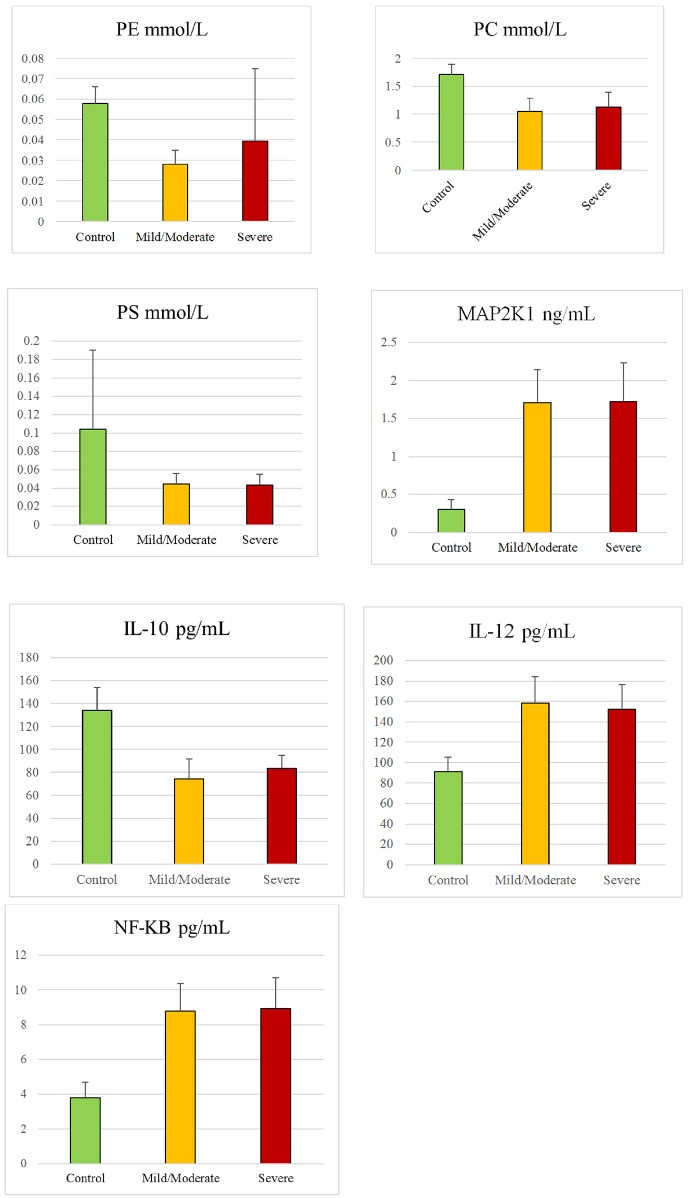
Set 1 biomarker data collected from 35 autistic patients and 38 healthy control participants. PE: phosphatidylethanolamine, PS: phosphatidylserine, PC: phosphatidylcholine, MAP2K1: mitogen-activated protein kinase kinase 1, IL-10: interleukin 10, IL-12: interleukin 12, NF-κB: nuclear factor kappa B.

**Fig 2:**
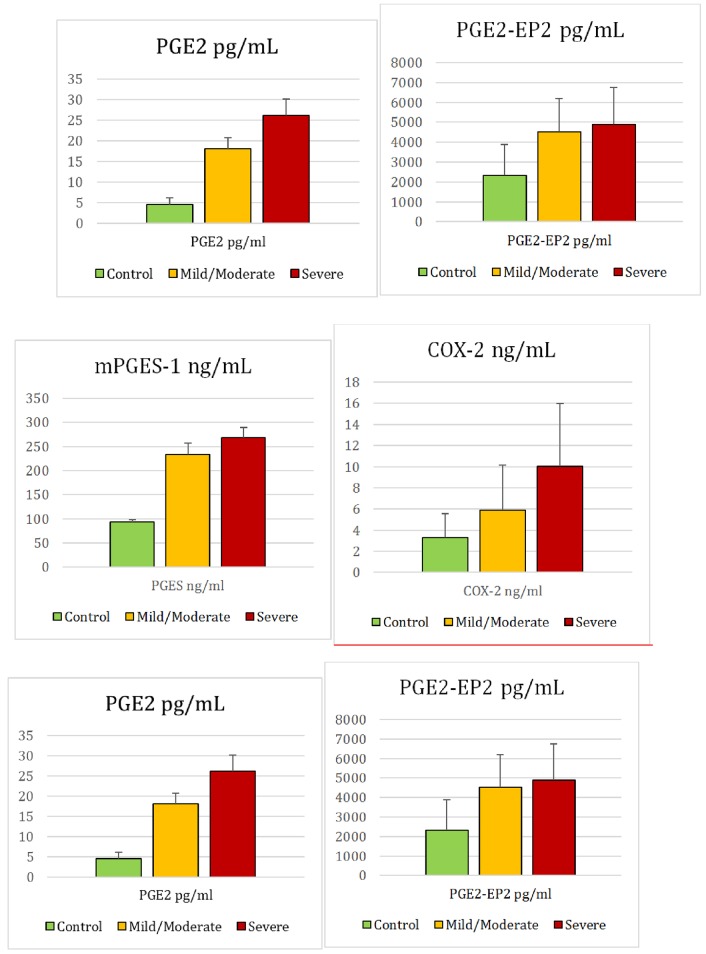
Set 2 biomarker data collected from 29 autistic patients and 16 healthy control participants. PGE2: prostaglandin E_2_, PGE2-EP2: prostaglandin E_2_ receptor 2, mPGES-1: membrane-bound prostaglandin E synthase 1, COX-2: cyclooxygenase 2, cPLA2: cytosolic phospholipase A2.

**Fig 3:**
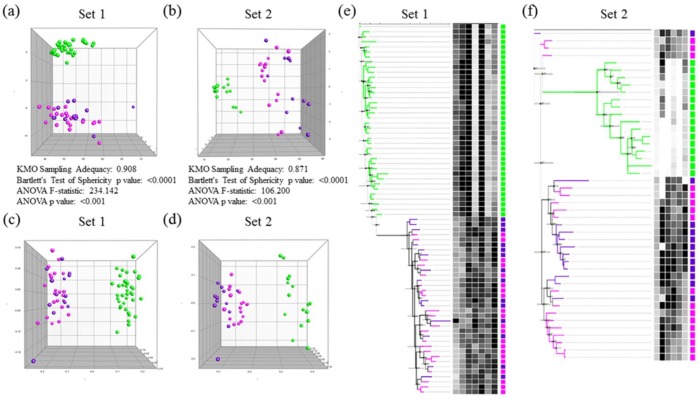
Separation of autistic and healthy control participants based on two sets of biomarkers. Principal component analysis (a and b) and multidimensional scaling (c and d) scatter plots based on set 1 (a and c) and set 2 biomarkers (b and d) show complete separation of autistic and healthy control groups. Hierarchical clustering shows efficient separation of autistic and healthy control groups based on set 1 (e) or 2 (f) biomarkers. Dendrograms were constructed from Canberra distances data using Neighbor joining algorithm. Heat maps depict marker values with darker grey indicative of higher values. Heat map variables from left to right are (e) PE, PS, PC, MAP2K1, IL-10, IL-12, and NFκB, and (f) PGE2, PGE2-EP2, PGES, cPLA2, 8-isoprostane, and COX-2. Autistic subjects with mild to moderate disease, those with severe disease, and control subjects are indicated with magenta, purple, and green squares, respectively.

**Fig 4:**
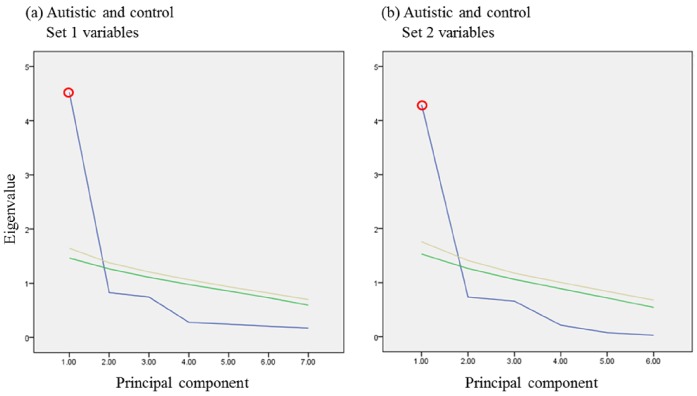
Determination of statistical significance of components in principal component analysis using Monte Carlo simulation. Set 1 (a) and 2 (b) data obtained from autistic and healthy control participants were analyzed with PCA. Screen plots show Eigen values of raw data (blue), as well as the 50^th^ (green) and 95^th^ percentile (yellow) simulated data. A principal component was considered statistically significant (circled in red) whenever its raw data Eigen value lay above the corresponding 95^th^ percentile simulated data Eigen value.

Two sets of biomarkers were tested: set 1 included 7, while set 2 included 6 biomarkers. For set 1, data were collected from 35 autistic and 38 healthy control participants, while for set 2, data were collected from 29 out of the same 35 autistic and 16 additional healthy participants. Among the 35 autistic patients, 21 were classified as having mild to moderate autism based on their sensory profiles, and the remaining 14 were considered to have severe autism (Figs 1 and 2). PCA, MDS, and hierarchical clustering were employed for the initial exploration of natural partitioning of participants. Using either group of markers, complete separation between autistic and healthy controls was achieved (Fig 3). Applying PCA to both sets of biomarkers was appropriate with Bartlett’s test of sphericity p value < 0.0001 for both and KMO measure of sampling adequacy of 0.908 and 0.871 for sets 1 and 2, respectively (Fig 3a and 3b). Only the first PC was statistically significant in either case (Fig 1). The most important markers in discriminating between autistic and healthy controls were determined by ranking biomarkers by their contribution to the principal component whose coordinates is where most of the separation between the two groups occurred. This turned out to be PC2 and PC1 for set 1 and 2 of biomarkers, respectively (Fig 3a and 3b). Thus the most discriminatory biomarkers in set 1 were found to be MAP2K1, NFκB, PS, and IL-10 (Table 1), while the most discriminatory in set 2 were mPGES-1, 8-isoprostane, and PGE2 (Table 2). MDS and hierarchical clustering showed similar results, with both yielding complete separation between autistic and controls using either set of markers (Fig 3c–3f). It is noteworthy that using set 2 data, the separation between autistic and controls was vastly on the PC2 coordinate, which did not meet statistical significance (Fig 4). However, complete separation between the two groups was supported by the results of other methods (MDS and hierarchical clustering).

**Table 1:** Contributions of variables (markers) to the first and second principal components (PC1 and PC2) in principal component analysis of 35 autistic and 38 healthy control subjects. Data were collected for a set of 7 markers; PE, PS, PC, MAP2K1, IL-10, IL-12, and NF-κB (set 1). Separation between autistic and control groups was mostly on PC2 coordinate. The most discriminatory variables are shown in boldface. The portion of variance explained by each principal component is shown between parentheses. * indicates a statistically significant principal component.

PC1* (84.7%)		PC2 (11.8%)	
PC	8.2	**PS**	**2.8**
IL-12	8.2	**IL-10**	**2.3**
IL-10	8.1	PE	2.2
PS	7.9	PC	2.0
PE	7.9	IL-12	-2.1
NFKB	7.8	**NFKB**	**-3.1**
MAP2K1	6.8	**MAP2K1**	**-4.9**

**Table 2:** Variable (marker) contributions to the first and second principal components (PC1 and PC2) in principal component analysis of 29 autistic and 16 healthy control subjects. Data were collected for a set of 6 markers; PGE2, PGE2-EP2, PGES, COX-2, cPLA2, and 8-isoprostane (set 2). In this analysis, autistic and control groups were mostly separated on PC1 coordinate. The most discriminatory variables are shown in boldface. The portion of variance explained by each principal component is shown between parentheses. * indicates a statistically significant principal component.

PC1* (90.9%)		PC2 (4.3%)	
**mPGES-1**	**6.64**	cPLA2	0.76
**8-Isoprostane**	**6.63**	PGE2-EP2	0.71
**PGE2**	**6.60**	8-Isoprostane	0.59
cPLA2	6.30	mPGES-1	0.48
PGE2-EP2	6.22	PGE2	0.33
COX-2	5.94	COX-2	-3.12

### Autistic patients with severely impaired sensory profiles can be separated from those with mild or moderate impairment based on a set of 6 markers

**Fig 5:**
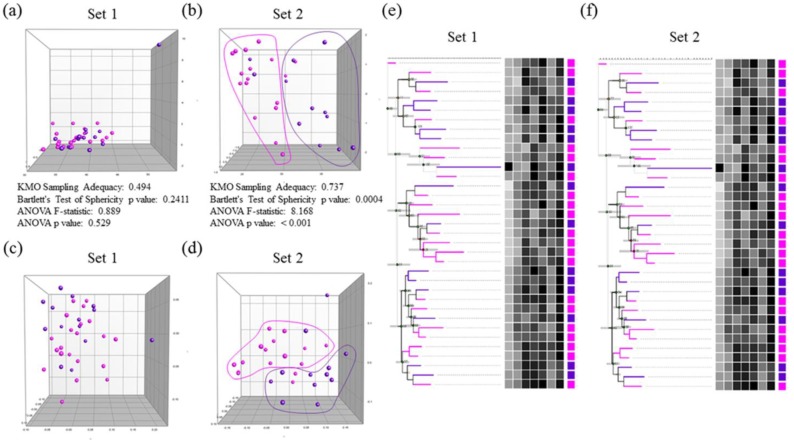
Separation of autistic patience based on the severity of sensory profile impairment. Autistic patients were classified into two groups, one with severe impairment of sensory profiles (purple squares) and the other with mild or moderate impairment (magenta squares). Principal component analysis (a&b) and multidimensional scaling (c&d) scatter plots based on set 1 (a&c) do not show separation between groups, while set 2 biomarkers (b&d) show visually discernible—although not complete—separation. Hierarchical clustering failed to show separation of autistic patient groups based on either set 1 (e) or 2 (f) biomarkers. Dendrograms were constructed from Canberra distances data using Neighbor joining algorithm. Heat maps depict marker values with darker grey indicative of higher values. Heat map variables from left to right are (e) PE, PS, PC, MAP2K1, IL-10, IL-12, and NFκB, and (f) PGE2, PGE2-EP2, PGES, cPLA2, 8-isoprostane, and COX-2.

**Fig 6:**
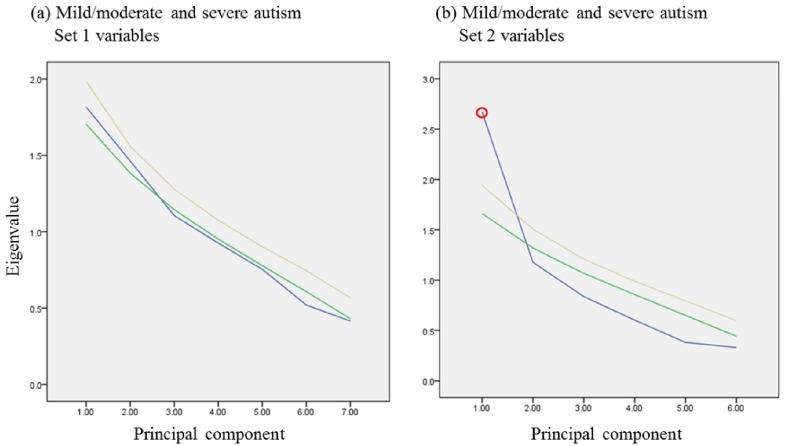
Determination of statistical significance of components in principal component analysis using Monte Carlo simulation. Set 1 (a) and 2 (b) data obtained from mild/moderate and severe autism groups were analyzed with PCA. Scree plots show eigenvalues of raw data (blue), as well as the 50^th^ (green) and 95^th^ percentile (yellow) simulated data. A principal component was considered statistically significant (circled in red) whenever its raw data eigenvalue lay above the corresponding 95^th^ percentile simulated data eigenvalue.

The next question addressed in this study was whether the two sets of biomarkers could be used to predict sensory profiles of autistic patients. Autistic patients were divided into a severe group, with sensory profiles ≤145, and a mild-to-moderate group, with sensory profiles >145. These two groups of autistic patients were not separable on the basis of set 1 biomarkers. Visual inspection of PCA, MDS, and hierarchical clustering inspection revealed no clear segregation between the groups. KMO test, Bartlett’s test, and ANOVA did not show statitically significant difference between the groups (Fig 5), and Monte Carlo simulation showed no statistically significant components (Fig 5a). Taken together, our data demonstrated that set 1 biomarkers were not able to segregate sensory profile groups among the autistic patients studied. Set 2 biomarkers, however, were able to segregate the two groups, which was apparent by visual inspection of PCA and MDS plots, although some overlap was observed. Analysis of variance revealed statistically significant difference between the groups, the use of PCA was shown to be appropriate for the dataset (KMO 0.737, Bartlett’s p value 0.0004), the first principal component (PC1) was statistically significant by Monte Carlo simulation, and the separation between the groups was predominantly on the PC1 coordinate (Fig 5). Combining the biomarkers of the two sets did not improve group separation (data not shown). Based variable contributions to PC1, PGES, 8-isoprostane, and PGE2 were the most discriminatory paramters among set 2 biomarkers (Table 3, Fig 6b).

**Table 3:** Variable (marker) contributions to the first and second principal components (PC1 and PC2) in principal component analysis of six markers tat corelated with sensory profiles of autistic patients.

PC1* (91.8%)		PC2 (4.5%)	
**PGES**	**5.34**	**PGE2-EP2**	**1.12**
**8-Isoprostane**	**5.34**	**8-Isoprostane**	**0.38**
**PGE2**	**5.31**	PGES	0.38
cPLA2	5.12	PGE2	0.20
PGE2-EP2	5.07	cPLA2	0.19
COX-2	4.76	**COX-2**	**-2.47**

**Fig 7:**
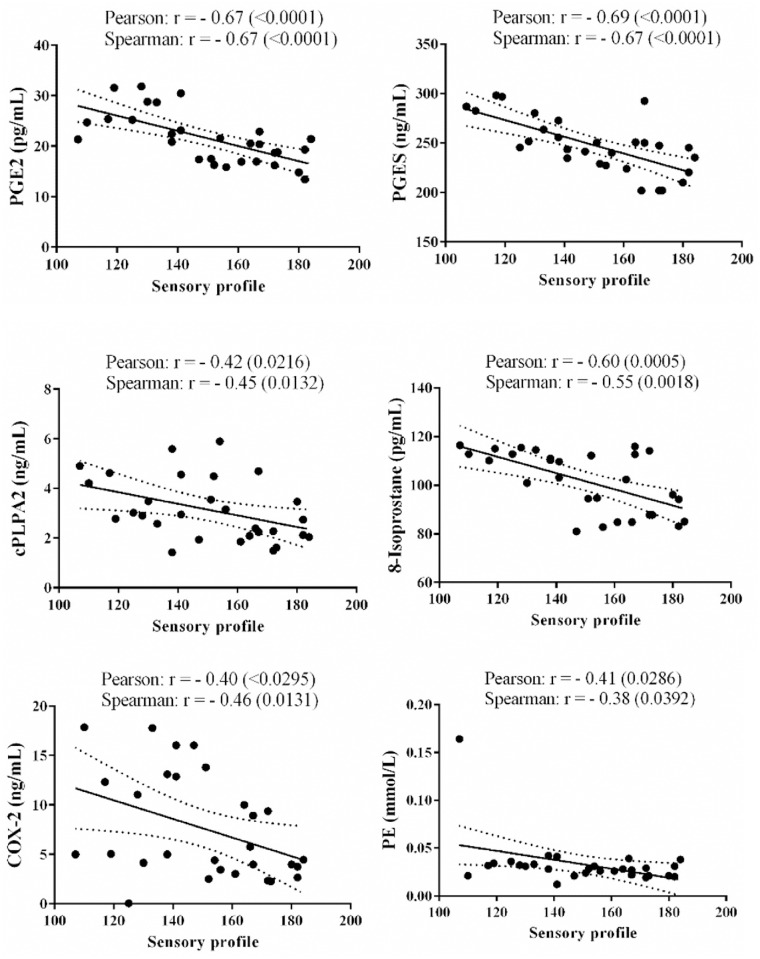
Correlation between sensory profiles of autistic patients and six markers. Correlation between sensory profile scores and each of the variables was calculated using Pearson and Spearman Correlation Coefficients to evaluate linear and monotonic nonlinear correlations, respectively. P values (in parenthesis) indicate the likelihood of obtaining the corresponding correlation value or a higher value due to random sampling alone. A linear regression line is shown with 95% confidence intervals (dotted lines).

Our exploratory studies using PCA and MDS showed separation between patients with severely impaired sensory profiles and those with mild to moderate impairment, which indictated that sensory profiles correlated with at least some of the variables. Correlation analysis revealed inverse linear correlation between sensory profiles and PE from set 1 biomarkers; and PGE2, PGES, 8-isoprostane, cPLA2, and COX-2 from set 2 biomarkers (Fig 7). To identify the most suitable biomarkers for disease severity prediction, stepwise multiple regression was performed. The linear combination of PGES, PGE2, and PE was significantly related to sensory profiles (F = 15.586 and p value = 0.000). The R value was 0.807 when using this linear combination, which indicated that approximately 65.2.% of sensory profile variance could be accounted for by the 3-marker combination (Table 4).

**Table 4:** Multiple regression analysis identifies a combination of 3 biomarkers as the best predictors of the degree of sensory profile impairment. Data of 29 autistic participants, including 17 with mild-to-moderate impairment and 12 with severe impairment.

Model	R	R Square	Adjusted R Square	F	p value
	0.689	0.474	0.455	24.380	0.000
mPGES-1, PGE2	0.767	0.588	0.556	18.559	0.000
mPGES-1, PGE2, PE	0.807	0.652	0.610	15.586	0.000

### Autism and the degree of sensory profile impairment associated with it can be pridicted using specific biomarkers

**Fig 8:**
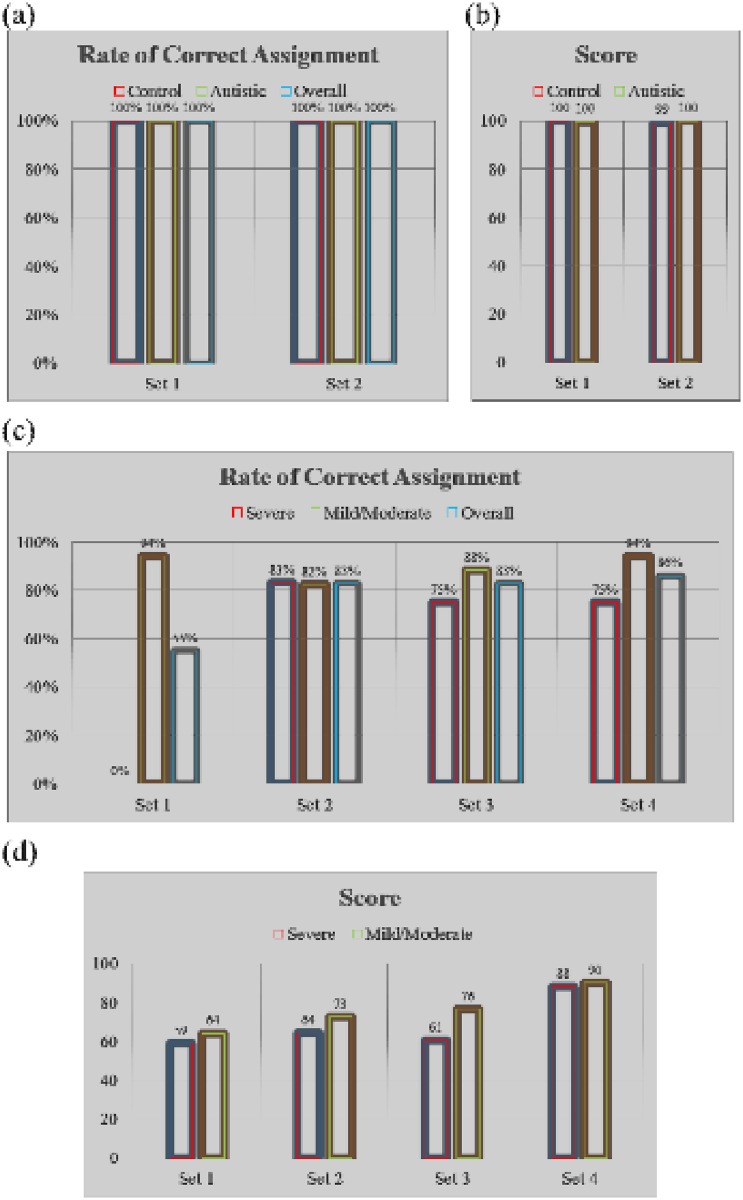
Rates of correct assignment in library-based identification. (a) Rates of correct assignment of autistic and control groups. Control and autistic library units were composed, respectively, of 39 and 35 participants when using set 1 biomarkers; and 16 and 29 participants when using set 2 biomarkers. (b) Rates of correct assignment in autstic patients with mild-to-moderate sensory profile impairment and those with high impairment. Severe and mild-to-moderate library units wre composed of 12 and 17 participants, respectively. Biomarkers used in the identification process were set 1: PE, PS, PC, MAP2K1, IL-10, IL-12, and NFκB; set 2: PGE2, PGE2-EP2, PGES, cPLA2, 8-isoprostane, and COX-2; set 3: PGE2, PGES, cPLA2, 8-isoprostane, COX-2, and PE; and set 4: PGE2, PGES, and PE.

To imperically predict the ability of biomarkers to identify autistic patients and the severity of sensory profile impairment, library-based identification was used. Library identification resulted in 100% correct assignments of both autistic and control participants based on either set 1 or 2 biomarkers, with very high assignment scores (Fig 8a & 8b). In terms of disease severity prediction and based on the results presented above, high rates of correct assignment were not predicted using set 1 biomarkers. However, identification using these markers was attempted to conifrm the predicted outcomes and to have data to compare with other sets of biomarkers. Using set 1 bionmakers, no correct assignments were made among particiapnts with severe impairments, while 94% were correctly assigned among the mild-to-moderate group, which cuminated in an overall rate of correct assignment of 55%. Using set 2, more than 80% of the participants were correctly assigned to their corresponding groups, with overall rate of correct assignment of 83%. We also tested identification using the 6 correlated biomarkers from sets 1 and 2 (Set 3: PGE2, mPGES-1, cPLA2, 8-isoprostane, COX-2, and PE) and the best predictors based on multiple regression analysis (Set 4: PGE2, PGES, and PE). Using set 3, the rates of correct assignment in the sever and mild-to-moderate groups were 75% and 88%, respectively, with an overall rate of correct assignment of 83%. The highest overall rate of correct assignment of 86% was achieved using set 4, with the rates of correct assignment in the severe and mild-to-moderate groups being 75% and 94%, respectively (Fig 8c). The highest assignment scores were also obatined using set 4 biomarkers (Fig 8d).

## Discussion

Clear separation between autistic patients and controls was demonstrated using PCA, and was confirmed using hierarchical clustering. In addition, autistic patients’ biomarker profiles could segregate them into at least two groups (Figs 3a–3f and 4). This observed clustering is based on the work of Hu and Steinberg [21], who showed that there could be four clusters within ASD regarding language deficits. They reported that while one group is characterized by severe language deficits, second group exhibited milder symptoms across language domains; the third showed noticeable savant skills, while the fourth group exhibited intermediate severity in language deficits but relatively lower frequency of savant skills. Moreover, the clustering seen within autistic participants of the present study (Fig 3a–3f) is in good agreement with the report of Subramanian (2015) which suggested that patients currently classified as autistic can differ in the abnormalities seen in major biochemical pathways that can result in either excessive or reduced synaptic connectivity in affected brain regions [22].

One of the markers tested in the current study is cytosolic phospholipase A2 (cPLA2), an enzyme that releases arachidonic acid (AA), a precursor of 2 series prostaglandins (PGs), from cell membrane phospholipid pool [23]. Children with autism are known to have higher phospholipase A2 (PLA2) activity compared to their matched control [24]. It has been proposed that instability observed in fatty acid levels may be as a result of an increase in PLA2 activity, perhaps in association with the high oxidative stress seen in autistic patients [25]. Different degrees of central nervous system (CNS) and gastrointestinal abnormalities reported in autistic patients [4,26,27] could be attributed to alterations in the COX-2/PGE2 pathway noted. Significant changes in the activity and expression of COX-2 induced by cytokines and other proinflammatory agents may result in alterations observed in immune responses seen in patients with autism. The strong correlation noted between sensory scores and COX-2 expression suggests that COX-2 metabolites may have a role in the function of sensory nerves and pain response [28] that, in turn, can be linked to their abnormalities seen in autistic children such as feeling annoyed when someone cuts their nails or hair, fearing from high places, close their eyes from strong light, put their hands on ears when there's high sound, etc. It is known that PGE2 can modulate the sensitivity of sensory nerves and functions as a mediator to induce fever [29]. PGE2, in turn, stimulates glutamate release from astrocytes and modulates the activity of neighboring neurons [30,31,32]. This may explain hyperactivity behavior, difficulty in subjugation to order and instruction that is related to severe sensory scores seen in autistic children. This is supported by the PCA- analysis of the first set of variables presented in Table 1 and the second set of COX-2/PGE2 [33] results presented in Table 2. Elevated levels of 8-isoprostane (8-iso-PGF2a) via the non-enzymatic oxidation of AA have been also detected in the urine of children with autism compared to healthy controls [34,35]).

The results presented in Fig 5(a)–5(f) along with those shown in Fig 2 suggest that PGE2, mPGES-1, 8-Isoprostane, COX-2, and cPLA2 are the best biomarkers among the candidate biomarkers tested for predicting high sensory abnormalities seen in autistic patients. This is confirmed by the clustering seen within the autistic patients when applying principal components analysis for the 1^st^ patch of variables. The association between COX-2/PGE2 signaling and sensory profile reported in the present study is confirmed by the clustering within autistic patients seen in Fig 3b. Using Dunn (2001) sensory processing, patients can be classified into four quadrants: Low Registration, Sensation Seeking, Sensation Sensitivity, and Sensation Avoidance. Low registration is a combination of high thresholds and passive responding. Sensation Seeking is a combination of high thresholds and active responding. Sensation Sensitivity is a combination of low thresholds and passive responding. Finally, Sensation Avoidance is a combination of low thresholds and active responding [36]

The significant negative correlations observed among the tested 6 lipid mediators and sensory profile (Fig 7) is supported by the high area under the curve, specificity and sensitivity recorded for these markers through the use of receiver operating characteristic (ROC) analysis (Unpublished Data). Role of the measured parameters in the etiology of abnormal sensory profile in autistic patients is supported by the work of Das [37,38], who suggested that an alteration in the metabolism of PUFAs plays a significant role in ASD. It was proposed that formation of excess of pro-inflammatory cytokines and eicosanoids derived from AA (such as PGE2, thromboxanes and leukotrienes) and simultaneously reduced formation of anti-inflammatory cytokines and anti-inflammatory bioactive lipids such as lipoxin A4 (LXA4) (from AA), resolvins (from EPA and DHA) and protectins (from DHA) may have a significant role in ASD. It was suggested that PUFAs and their anti-inflammatory metabolites enhance neurite outgrowth, promote neuronal survival, and modulate actions of neurotransmitters [37, 38]. It is likely that when there is an imbalance between pro- and anti-inflammatory metabolites of PUFAs and their precursors (between n-6 AA and n-3 EPA and DHA) it may result in oxidative stress that leads neuronal damage leading to the development of autism. In this context, estimation of plasma levels of isoprostane could be used as a marker of oxidative stress related to PUFAs and their metabolites. The contribution of isoprostane- related oxidative injury in autism can also be related to possible autoimmunity occurring in this disorder [39]. For example, plasma levels of 8-isoprostane have been correlated with anti-neural antibodies. Attack of self-proteins, modified by adduction of lipid-derived electrophiles generated by oxidative injury as been documented in autistic patients [40,41]. The reported decrease of IL-10, an anti-inflammatory cytokine, and an increase in IL-12, a pro-inflammatory cytokine, reported in the present study are in support of the evidence that neuronal inflammation is involved in the pathogenesis of autism. Two recent studies demonstrated that children with autism displayed altered immune profiles and function, characterized by a systemic deficit of Foxp3, a transcription factor responsible for regulation and differentiation of T regulatory (Treg) cells and increased dendritic cells, major producers of IL-10 and IL-12 respectively [41,42]. Moreover, the recently recorded association of impaired lipid metabolism, increase in pro-inflammatory cytokines and oxidative stress and altered nutritional status in the etiopathology of autism [43] are in support of the results presented here. Thus, measurement of the plasma levels of lipid peroxides, which is reflected by 8-isoprostane concentrations in biological fluids, may help to identify autistic patients who are more likely to be benefited from antioxidant treatments.

## Conclusion

The present study demonstrated that identification of autistic patients based on biomarkers is feasible. Based on these biomarkers, the level of sensory profile impairment among autistic patients could be predicted with impressive success. The results suggest that accurately predicting the severity of autism using these biomarkers is possible. Studies on much large number of patients are necessary to confirm the validity of these conclusions.

## Supporting Information

S1 Table Set 1 biomarker data collected from 35 autistic patients and 38 healthy control participants. PE: phosphatidylethanolamine, PS: phosphatidylserine, PC: phosphatidylcholine, MAP2K1: mitogen-activated protein kinase kinase 1, IL-10: interleukin 10, IL-12: interleukin 12, NF-κB: nuclear factor kappa B.
(DOC)

S2 Table Set 2 biomarker data collected from 29 autistic patients and 16 healthy control participants. PGE2: prostaglandin E_2_, PGE2-EP2: prostaglandin E_2_ receptor 2, PGES: membrane-bound prostaglandin E synthase 1, COX-2: cyclooxygenase 2, cPLA2: cytosolic phospholipase A2.
(DOC)

## Abbreviations

AA: arachidonic acid
CARS: Childhood autism rating scale
COX-2: cyclooxygenase-2
cPLA2: cytosolic phospholipase A2
IL-1: interleukin-1
IL- 10: interleukin-10
mPGES-1: microsomal prostaglandin synthase-1
MAP2K1: mitogen-activated protein kinase kinase 1
NFκB: nuclear factor-κappa B
PE: phosphatidyl ethanolamine
PC: phosphatidyl choline
PS: phosphatidyl serine
PC1: First principal component
PC2: Second principal component
PGE2: prostaglandin E2
PGE2-EP2: prostaglandin E2-EP2 receptors
PUFAs: polyunsaturated fatty acids
ROC-curve: receiver operating characteristics curve
ROS: reactive oxygen species
SRS: social responsiveness scale
SSP: short sensory profile
