# Supplementary material for: Identification of Biomarkers of Impaired Sensory Profiles among Autistic Patients
Source: PLoS One. 2016 Nov 8;11(11):e0164153. doi: 10.1371/journal.pone.0164153 (PMC5100977; doi:10.1371/journal.pone.0164153)
Supplement: S1 Table — PE: phosphatidylethanolamine, PS: phosphatidylserine, PC: phosphatidylcholine, MAP2K1: mitogen-activated protein kinase kinase 1, IL-10: interleukin 10, IL-12: interleukin 12, NF-κB: nuclear factor kappa B. (DOC) [file pone.0164153.s001.doc]

**S1 Table.** Set 1 biomarker data collected from 35 autistic patients and 38 healthy control participants. PE: phosphatidylethanolamine, PS: phosphatidylserine, PC: phosphatidylcholine, MAP2K1: mitogen-activated protein kinase kinase 1, IL-10: interleukin 10, IL-12: interleukin 12, NF-κB: nuclear factor kappa B.

| Participant ID | Sensory Profile Score | Disease Grade | PE mmol/L | PS mmol/L | PC mmol/L | MAP2K1 ng/mL | IL-10 pg/mL | IL-12 pg/mL | NF-KB pg/mL |
| --- | --- | --- | --- | --- | --- | --- | --- | --- | --- |
| 2 | 133 | Severe | 0.03 | 0.05 | 1.44 | 1.13 | 97.47 | 179.57 | 7.78 |
| 3 | 117 | Severe | 0.03 | 0.06 | 0.70 | 1.63 | 82.17 | 172.26 | 9.63 |
| 4 | 151 | Mild/Moderate | 0.02 | 0.04 | 1.19 | 1.87 | 53.69 | 192.15 | 8.70 |
| 5 | 152 | Mild/Moderate | 0.03 | 0.06 | 0.97 | 1.69 | 67.23 | 150.08 | 9.65 |
| 7 | 164 | Mild/Moderate | 0.03 | 0.04 | 0.97 | 1.05 | 80.77 | 155.21 | 11.82 |
| 10 | 154 | Mild/Moderate | 0.03 | 0.06 | 1.04 | 1.45 | 84.52 | 122.82 | 8.96 |
| 13 | 167 | Mild/Moderate | 0.03 | 0.04 | 0.41 | 1.99 | 58.03 | 145.10 | 6.21 |
| 16 | 166 | Mild/Moderate | 0.04 | 0.05 | 1.29 | 2.34 | 103.27 | 138.99 | 9.37 |
| 17 | 172 | Mild/Moderate | 0.03 | 0.04 | 1.00 | 1.78 | 96.80 | 134.26 | 8.40 |
| 23 | 161 | Mild/Moderate | 0.03 | 0.07 | 0.93 | 1.92 | 52.91 | 137.99 | 8.68 |
| 24 | 110 | Severe | 0.02 | 0.04 | 1.14 | 1.28 | 69.56 | 204.88 | 9.22 |
| 26 | 173 | Mild/Moderate | 0.02 | 0.03 | 1.22 | 1.73 | 48.87 | 207.38 | 6.46 |
| 27 | 154 | Mild/Moderate | 0.03 | 0.04 | 1.31 | 1.78 | 81.86 | 170.65 | 9.77 |
| 29 | 133 | Severe | 0.03 | 0.05 | 1.34 | 2.19 | 84.76 | 157.31 | 7.31 |
| 32 | 130 | Severe | 0.03 | 0.06 | 1.35 | 1.54 | 97.12 | 147.17 | 10.16 |
| 42 | 138 | Severe | 0.04 | 0.04 | 1.16 | 1.22 | 95.09 | 178.48 | 6.79 |
| 44 | 167 | Mild/Moderate | 0.02 | 0.05 | 1.07 | 0.75 | 57.78 | 155.12 | 10.81 |
| 46 | 141 | Severe | 0.01 | 0.05 | 1.16 | 1.85 | 86.16 | 122.34 | 7.48 |
| 48 | 119 | Severe | 0.03 | 0.05 | 1.10 | 1.62 | 78.59 | 147.87 | 9.84 |
| 49 | 138 | Severe | 0.03 | 0.03 | 1.20 | 1.07 | 92.44 | 118.10 | 10.97 |
| 50 | 182 | Mild/Moderate | 0.03 | 0.05 | 1.32 | 1.90 | 83.26 | 196.70 | 9.24 |
| 51 | 172 | Mild/Moderate | 0.02 | 0.04 | 0.93 | 1.17 | 85.63 | 183.87 | 8.82 |
| 52 | 184 | Mild/Moderate | 0.04 | 0.04 | 0.80 | 1.94 | 48.30 | 173.59 | 8.62 |
| 53 | 182 | Mild/Moderate | 0.02 | 0.03 | 1.22 | 1.11 | 71.59 | 155.32 | 6.88 |
| 54 | 125 | Severe | 0.04 | 0.04 | 1.29 | 2.21 | 75.94 | 132.60 | 7.08 |
| 63 | 180 | Mild/Moderate | 0.02 | 0.04 | 0.99 | 1.57 | 106.91 | 145.82 | 8.52 |
| 69 | 156 | Mild/Moderate | 0.03 | 0.06 | 1.27 | 2.11 | 89.33 | 152.26 | 9.91 |
| 72 | 159 | Mild/Moderate | 0.03 | 0.03 | 1.39 | 1.34 | 71.13 | 122.49 | 8.72 |
| 75 | 101 | Severe | 0.03 | 0.05 | 1.07 | 2.59 | 78.12 | 136.99 | 10.93 |
| 97 | 141 | Severe | 0.04 | 0.03 | 0.92 | 1.35 | 98.02 | 145.54 | 11.91 |
| 127 | 107 | Severe | 0.16 | 0.04 | 0.50 | 2.58 | 67.92 | 154.15 | 9.74 |
| 132 | 147 | Mild/Moderate | 0.02 | 0.05 | 1.08 | 2.01 | 76.41 | 161.10 | 7.35 |
| 134 | 168 | Mild/Moderate | 0.04 | 0.04 | 0.69 | 2.47 | 66.21 | 199.09 | 11.63 |
| 148 | 128 | Severe | 0.03 | 0.02 | 1.39 | 1.88 | 64.67 | 140.54 | 6.32 |
| 205 | 190 | Mild/Moderate | 0.03 | 0.03 | 0.97 | 1.88 | 81.01 | 125.80 | 6.18 |
| C10 |  |  | 0.06 | 0.14 | 1.64 | 0.56 | 124.44 | 96.98 | 4.14 |
| C11 |  |  | 0.05 | 0.09 | 1.92 | 0.32 | 121.96 | 81.86 | 4.36 |
| C11379 |  |  | 0.05 | 0.09 | 1.63 | 0.20 | 144.15 | 109.86 | 3.20 |
| C11502 |  |  | 0.06 | 0.09 | 1.78 | 0.26 | 140.15 | 85.42 | 3.56 |
| C11586 |  |  | 0.05 | 0.07 | 1.80 | 0.26 | 120.80 | 73.65 | 4.98 |
| C11593 |  |  | 0.06 | 0.06 | 1.66 | 0.22 | 196.10 | 83.17 | 3.90 |
| C11594 |  |  | 0.05 | 0.10 | 1.95 | 0.22 | 163.84 | 100.57 | 4.95 |
| C11597 |  |  | 0.06 | 0.08 | 1.75 | 0.34 | 122.03 | 99.25 | 4.14 |
| C11694 |  |  | 0.06 | 0.10 | 2.17 | 0.37 | 139.30 | 98.51 | 2.87 |
| C11736 |  |  | 0.05 | 0.09 | 1.49 | 0.20 | 165.03 | 83.60 | 2.79 |
| C11785 |  |  | 0.05 | 0.10 | 1.87 | 0.22 | 131.30 | 90.52 | 3.09 |
| C13 |  |  | 0.05 | 0.10 | 1.73 | 0.20 | 115.53 | 102.49 | 2.40 |
| C15 |  |  | 0.06 | 0.12 | 1.88 | 0.18 | 129.58 | 79.90 | 5.13 |
| C16 |  |  | 0.05 | 0.09 | 1.45 | 0.37 | 133.72 | 82.81 | 2.87 |
| C17 |  |  | 0.06 | 0.09 | 1.47 | 0.29 | 129.31 | 80.21 | 3.19 |
| C20 |  |  | 0.06 | 0.61 | 1.85 | 0.28 | 122.02 | 115.05 | 3.63 |
| C21 |  |  | 0.05 | 0.08 | 1.55 | 0.29 | 120.25 | 108.10 | 4.03 |
| C22 |  |  | 0.07 | 0.08 | 2.05 | 0.23 | 145.27 | 81.17 | 2.71 |
| C24 |  |  | 0.06 | 0.07 | 1.76 | 0.15 | 167.57 | 90.21 | 3.00 |
| C25 |  |  | 0.06 | 0.08 | 1.75 | 0.12 | 111.50 | 143.07 | 4.11 |
| C27 |  |  | 0.07 | 0.11 | 1.67 | 0.20 | 148.14 | 106.97 | 5.41 |
| C28 |  |  | 0.08 | 0.13 | 1.31 | 0.53 | 129.12 | 84.84 | 2.84 |
| C29 |  |  | 0.07 | 0.11 | 1.54 | 0.34 | 110.19 | 79.26 | 2.54 |
| C3 |  |  | 0.06 | 0.09 | 1.71 | 0.46 | 116.12 | 85.88 | 5.23 |
| C32 |  |  | 0.07 | 0.08 | 1.64 | 0.23 | 125.88 | 104.49 | 4.05 |
| C33 |  |  | 0.06 | 0.08 | 1.60 | 0.74 | 108.74 | 80.09 | 4.48 |
| C33711 |  |  | 0.05 | 0.07 | 2.09 | 0.23 | 105.37 | 81.15 | 4.12 |
| C34 |  |  | 0.07 | 0.08 | 1.47 | 0.22 | 120.88 | 71.80 | 4.53 |
| C36 |  |  | 0.06 | 0.11 | 1.46 | 0.33 | 161.27 | 78.15 | 2.78 |
| C37 |  |  | 0.06 | 0.08 | 1.68 | 0.41 | 152.91 | 86.52 | 3.75 |
| C38 |  |  | 0.06 | 0.08 | 1.59 | 0.22 | 143.29 | 87.52 | 3.02 |
| C39 |  |  | 0.05 | 0.08 | 1.69 | 0.19 | 111.89 | 78.40 | 4.72 |
| C4 |  |  | 0.05 | 0.08 | 1.68 | 0.50 | 169.17 | 82.26 | 4.91 |
| C40 |  |  | 0.06 | 0.08 | 1.72 | 0.31 | 116.41 | 105.80 | 2.94 |
| C5 |  |  | 0.05 | 0.10 | 1.85 | 0.28 | 133.84 | 76.43 | 4.39 |
| C7 |  |  | 0.06 | 0.08 | 1.78 | 0.25 | 121.34 | 100.91 | 5.02 |
| C8 |  |  | 0.04 | 0.10 | 1.75 | 0.24 | 134.14 | 94.79 | 2.90 |
| C9 |  |  | 0.05 | 0.07 | 1.58 | 0.41 | 142.25 | 84.73 | 3.63 |
