# Supplementary material for: Identification of Biomarkers of Impaired Sensory Profiles among Autistic Patients
Source: PLoS One. 2016 Nov 8;11(11):e0164153. doi: 10.1371/journal.pone.0164153 (PMC5100977; doi:10.1371/journal.pone.0164153)
Supplement: S2 Table — PGE2: prostaglandin E2, PGE2-EP2: prostaglandin E2 receptor 2, PGES: membrane-bound prostaglandin E synthase 1, COX-2: cyclooxygenase 2, cPLA2: cytosolic phospholipase A2. (DOC) [file pone.0164153.s002.doc]

**S2 Table.** Set 2 biomarker data collected from 29 autistic patients and 16 healthy control participants. PGE2: prostaglandin E_2_, PGE2-EP2: prostaglandin E_2_ receptor 2, PGES: membrane-bound prostaglandin E synthase 1, COX-2: cyclooxygenase 2, cPLA2: cytosolic phospholipase A2.

| Participant ID | Sensory Profile Score | Disease Grade | PGE2 pg/ml | PGE2-EP2 pg/ml | mPGES-1 ng/ml | COX-2 ng/ml | cPLA2 ng/ml | 8-Isoprostane pg/ml |
| --- | --- | --- | --- | --- | --- | --- | --- | --- |
| 3 | 117 | Severe | 25.37 | 4979.03 | 298.27 | 12.32 | 4.63 | 110.14 |
| 4 | 151 | Mild/Moderate | 17.49 | 1450.20 | 250.35 | 13.80 | 3.55 | 94.48 |
| 5 | 152 | Mild/Moderate | 16.30 | 4894.90 | 228.83 | 2.51 | 4.49 | 112.24 |
| 7 | 164 | Mild/Moderate | 20.53 | 4752.04 | 250.58 | 10.01 | 2.09 | 102.35 |
| 13 | 167 | Mild/Moderate | 22.87 | 3917.21 | 250.16 | 8.93 | 2.25 | 115.97 |
| 16 | 166 | Mild/Moderate | 16.97 | 4783.25 | 201.86 | 5.75 | 2.39 | 84.81 |
| 17 | 172 | Mild/Moderate | 18.68 | 5767.59 | 201.86 | 2.34 | 1.49 | 87.77 |
| 23 | 161 | Mild/Moderate | 16.90 | 6919.43 | 223.84 | 3.03 | 1.86 | 84.80 |
| 24 | 110 | Severe | 24.71 | 4984.99 | 282.51 | 17.85 | 4.21 | 112.83 |
| 26 | 173 | Mild/Moderate | 18.82 | 6019.64 | 201.97 | 2.28 | 1.61 | 87.77 |
| 27 | 154 | Mild/Moderate | 21.59 | 3816.86 | 227.19 | 4.40 | 5.90 | 94.71 |
| 29 | 133 | Severe | 28.69 | 7326.43 | 263.61 | 17.79 | 2.58 | 114.55 |
| 32 | 130 | Severe | 28.80 | 5702.40 | 280.25 | 4.14 | 3.49 | 100.87 |
| 42 | 138 | Severe | 20.85 | 4623.56 | 255.64 | 4.99 | 1.42 | 110.39 |
| 44 | 167 | Mild/Moderate | 20.38 | 5486.04 | 292.40 | 3.97 | 4.70 | 112.70 |
| 46 | 141 | Severe | 23.17 | 5058.23 | 234.52 | 12.88 | 2.95 | 103.11 |
| 48 | 119 | Severe | 31.59 | 8635.27 | 296.72 | 5.05 | 2.78 | 115.03 |
| 49 | 138 | Severe | 22.42 | 4950.74 | 272.54 | 13.12 | 5.59 | 111.02 |
| 50 | 182 | Mild/Moderate | 13.45 | 7864.09 | 220.32 | 2.65 | 2.75 | 94.25 |
| 51 | 172 | Mild/Moderate | 16.23 | 1681.17 | 247.27 | 9.38 | 2.27 | 114.16 |
| 52 | 184 | Mild/Moderate | 21.42 | 2915.30 | 235.31 | 4.45 | 2.04 | 85.06 |
| 53 | 182 | Mild/Moderate | 19.31 | 3903.00 | 245.03 | 3.73 | 2.13 | 83.17 |
| 54 | 125 | Severe | 25.23 | 2021.55 | 245.41 | 0.05 | 3.02 | 112.85 |
| 63 | 180 | Mild/Moderate | 14.82 | 3295.81 | 210.16 | 3.97 | 3.47 | 96.11 |
| 69 | 156 | Mild/Moderate | 15.85 | 4544.49 | 239.96 | 3.44 | 3.16 | 82.80 |
| 97 | 141 | Severe | 30.49 | 2547.78 | 243.50 | 16.04 | 4.56 | 109.69 |
| 127 | 107 | Severe | 21.36 | 2766.78 | 286.87 | 5.00 | 4.91 | 116.42 |
| 132 | 147 | Mild/Moderate | 17.38 | 4965.61 | 241.17 | 16.04 | 1.94 | 80.94 |
| 148 | 128 | Severe | 31.84 | 4955.20 | 251.61 | 11.04 | 2.90 | 115.52 |
| C11157 |  |  | 7.05 | 3742.61 | 96.27 | 2.27 | 0.16 | 22.97 |
| C11207 |  |  | 6.82 | 4278.83 | 99.11 | 7.19 | 0.13 | 23.51 |
| C11218 |  |  | 7.26 | 4987.98 | 96.49 | 8.64 | 0.25 | 27.33 |
| C11258 |  |  | 3.45 | 950.65 | 92.20 | 1.60 | 0.13 | 24.17 |
| C11259 |  |  | 1.46 | 877.70 | 91.51 | 1.68 | 0.60 | 22.22 |
| C11343 |  |  | 2.91 | 905.39 | 95.29 | 2.19 | 0.11 | 21.68 |
| C11354 |  |  | 6.19 | 3162.41 | 87.80 | 3.39 | 0.13 | 25.00 |
| C11512 |  |  | 4.29 | 484.29 | 90.62 | 2.00 | 1.12 | 27.03 |
| C11519 |  |  | 4.82 | 868.84 | 92.89 | 2.33 | 0.24 | 26.16 |
| C11527 |  |  | 4.15 | 3686.57 | 93.40 | 6.85 | 0.48 | 25.38 |
| C11538 |  |  | 2.67 | 971.22 | 88.00 | 2.26 | 0.36 | 23.89 |
| C11570 |  |  | 5.54 | 2671.70 | 94.98 | 2.33 | 0.12 | 25.19 |
| C11656 |  |  | 4.57 | 1183.48 | 103.33 | 1.57 | 0.40 | 24.63 |
| C11683 |  |  | 4.52 | 3609.22 | 95.84 | 1.55 | 0.80 | 28.56 |
| C11734 |  |  | 3.41 | 890.61 | 85.85 | 3.34 | 0.34 | 23.62 |
| C11848 |  |  | 3.42 | 4040.90 | 92.88 | 3.84 | 0.54 | 25.96 |
